# Control strategies for inverted pendulum: A comparative analysis of linear, nonlinear, and artificial intelligence approaches

**DOI:** 10.1371/journal.pone.0298093

**Published:** 2024-03-07

**Authors:** Saqib Irfan, Liangyu Zhao, Safeer Ullah, Adeel Mehmood, Muhammad Fasih Uddin Butt

**Affiliations:** 1 School of Aerospace Engineering, Beijing Institute of Technology, Beijing, China; 2 Department of Electrical Engineering, Quaid-e-Azam College of Engineering & Technology, Sahiwal, Pakistan; 3 School of Computer Science, Faculty of Science and Engineering, University of Hull, Hull, United Kingdom; 4 Department of Electrical and Computer Engineering, COMSATS University Islamabad, Islamabad, Pakistan; University of Shanghai for Science and Technology, CHINA

## Abstract

An inverted pendulum is a challenging underactuated system characterized by nonlinear behavior. Defining an effective control strategy for such a system is challenging. This paper presents an overview of the IP control system augmented by a comparative analysis of multiple control strategies. Linear techniques such as linear quadratic regulators (LQR) and progressing to nonlinear methods such as Sliding Mode Control (SMC) and back-stepping (BS), as well as artificial intelligence (AI) methods such as Fuzzy Logic Controllers (FLC) and SMC based Neural Networks (SMCNN). These strategies are studied and analyzed based on multiple parameters. Nonlinear techniques and AI-based approaches play key roles in mitigating IP nonlinearity and stabilizing its unbalanced form. The aforementioned algorithms are simulated and compared by conducting a comprehensive literature study. The results demonstrate that the SMCNN controller outperforms the LQR, SMC, FLC, and BS in terms of settling time, overshoot, and steady-state error. Furthermore, SMCNN exhibit superior performance for IP systems, albeit with a complexity trade-off compared to other techniques. This comparative analysis sheds light on the complexity involved in controlling the IP while also providing insights into the optimal performance achieved by the SMCNN controller and the potential of neural network for inverted pendulum stabilization.

## 1 Introduction

The design of a control system for an inverted pendulum (IP) is a classical problem employed in nonlinear control systems. IP has many practical applications in various fields, such as humanoid robots and Segways. IP is a highly unstable and nonlinear system with a very complex nature. As an under-actuated system, the control design of an IP is considered a challenging task.

Several types of inverted pendulums, such as rotational IP and pendulum on the cart, have been previously tested, and researchers have proposed various methods to control these IP systems. The authors of [1–3] proposed a backstepping (BS)-based control technique for IP control. They proposed that the BS controls a two-step approach where swing up, while upward balancing is attained by a linear integral regulator. Lee et al. (2015) proposed an output feedback-based technique in the existence of uncertainties to stabilize IP on a cart [4]. A high-gain observer is used to estimate the states that are not measured in order to combat their uncertain nature. Lee and Takangi (1993) proposed an optimized genetic algorithm through a fuzzy controller to control the IP [5]. The genetic algorithm methodologies in control system engineering have been applied to several problems. Cuevas et al. (2015) proposed a fuzzy logic-based optimal controller for IP, in which the results for both the phase plan and linguistic trajectories are presented, and they demonstrated stable characteristics [6]. Optimal PID control for the linear model of IP is combined with pole placement algorithms to obtain the performance specifications, which leads to firefly optimization control [7]. Similarly, Eltohamy and Kuo (1998) designed a nonlinear controller for a single IP, based on an unstable upright position. An extended state observer is designed to observe the disturbances and uncertainties that have a rejection ability [8]. The researcher in [9] experimented with a traditional fuzzy controller to stabilize a single IP and improve the dynamics of the system accordingly.

Linear Quadratic Regulator (LQR) is a classical linear control system that can control those systems where disturbances and uncertainties are absent. This technique allows one to find the closed-loop gain location for the system by guaranteeing system stability in the presence of all states of the system [10, 11]. Sliding Mode Control (SMC) is a robust control technique that deals with the parametric uncertainties of matched and unmatched disturbances [12–14]. More consistency is required between the mathematical and actual models of the system. To overcome these discrepancies, robust control techniques such as SMC are more effective [15]. Fuzzy Logic Controller (FLC) is an artificial intelligence (AI) control technique that is used to develop a model for a complex system. This simplifies the model under certain assumptions and reduces the complexity of the system. It maintains the system’s energy in a steady state (up down position) [16, 17]. Similarly, a Neural Network (NN) is a practical algorithm for modeling nonlinear statistical scenarios by providing a method for logistic regression. It estimates the function, which has multiple inputs initially considered as unknown, and interconnects the system that exchanges information with each other [18]. NN connections have a numerical weight matrix, and based on previous information, NNs adapt to the input to achieve better learning capabilities [19–21].

This research study explores linear, nonlinear, and AI control strategies, such as LQR, SMC, BS, FLC, and SMCNN, and a comparative analysis is augmented with simulations of the selected algorithms. An IP without a controller is inherently unstable. Hence, to check and maintain its stability, we must manipulate it to check the response of the system vertically and horizontally. The FLC technique provides a benchmark for testing IP response without a mathematical approach. It stabilizes the system and maintains the cart in the desired position. The SMC is designed and implemented to check the response of the nonlinear and underactuated systems. It has a single input and two outputs for the cart position and pendulum angle. Therefore, this technique stabilizes the uncertain SIMO and MIMO systems. The LQR controller, which is an optimal control technique for the desired trajectories, is also simulated. The BS and SMCNN are explored in terms of IP stability to observe the behavior of the system. The FLC, SMC, and LQR simulation results are compared to analyze the behavior of the linear and nonlinear families on the control strategies.

The remainder of this paper is organized as follows. Section 1 describes the details of IP modeling, including linear and nonlinear models, while Section 2 presents different control approaches to stabilize the IP. Section 3 presents the results of the implemented control techniques, and Section 4 concludes the overall analysis of our research.

## 2 Modeling of inverted pendulum

**Fig 1:**
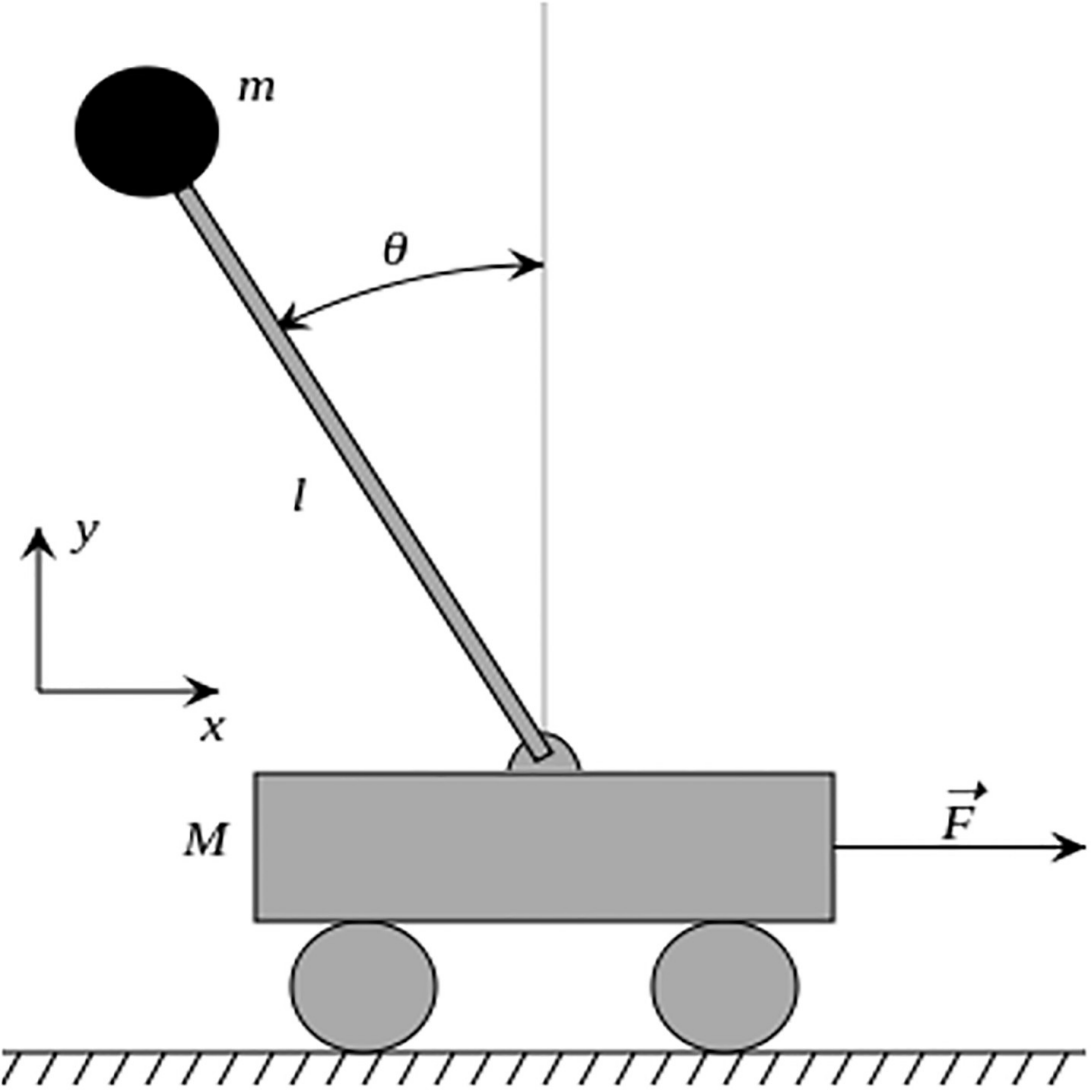
Physical model of an inverted pendulum.

The physical system of an IP is depicted in Fig 1, and comprises an IP mounted on a cart moving on a rail. The translational movement of the cart is enabled by DC motors that swing freely in a vertical position. A motor shaft is connected to the cart using thin steel wire. The IP system model is divided into two parts. The first is the mechanical structure of the cart and pendulum angle and the second is the DC motor transmission model.

The mathematical model of the IP is formulated using a Newtonian approach. The movement of the cart is due to horizontally applied forces, as it is not affected by vertical forces, and it receives the input through an actuator (DC motor) [22]. A nonlinear mathematical model of an IP is formulated using a Newtonian approach. The following equations describe the nonlinear mathematical model of an inverted pendulum.

$$\left(M+m\right)\ddot{x}+mL\ddot{\theta }\;\text{cos}\;\theta -ml\theta^{2}\;\text{sin}\;\theta +B\dot{x}=T$$ (1)

$$\begin{array}{c} \left(I+ml^{2}\right)\ddot{\theta }+mgl\;\text{sin}\;\theta =-ml\ddot{x}\;\text{cos}\;\theta \end{array}$$ (2)

Where *M* represents the cart mass, *m* is the mass of the Pendulum, *L* represents the cart length, *l* is the length of the pendulum, *B* is the coefficient of friction of the cart, *I* is the moment of inertia, *T* is the torque in the form of external input force which moves cart in the horizontal plane, *x* is the cart position while $\ddot{x}$ is the cart acceleration, *θ* is the angular position of the pendulum and $\ddot{\theta }$ is representing the angular acceleration. Eqs (1) and (2) are nonlinear and have been linearized to implement a linear controller. As *θ* is very small, it leads to the fact that the square of the derivative of *θ* is assumed to be zero. Hence, *θ*^2^ = 0, and the linearized mathematical model of the system can be expressed as follows:

$$\begin{array}{c} \left(M+m\right)\ddot{x}+B\;\dot{x}-mL\ddot{\theta }=Fa \end{array}$$ (3)

$$\begin{array}{c} \left(I+mL^{2}\right)\ddot{\theta }-mgL\theta =mL\ddot{x} \end{array}$$ (4)

## 3 Control techniques

**Fig 2:**
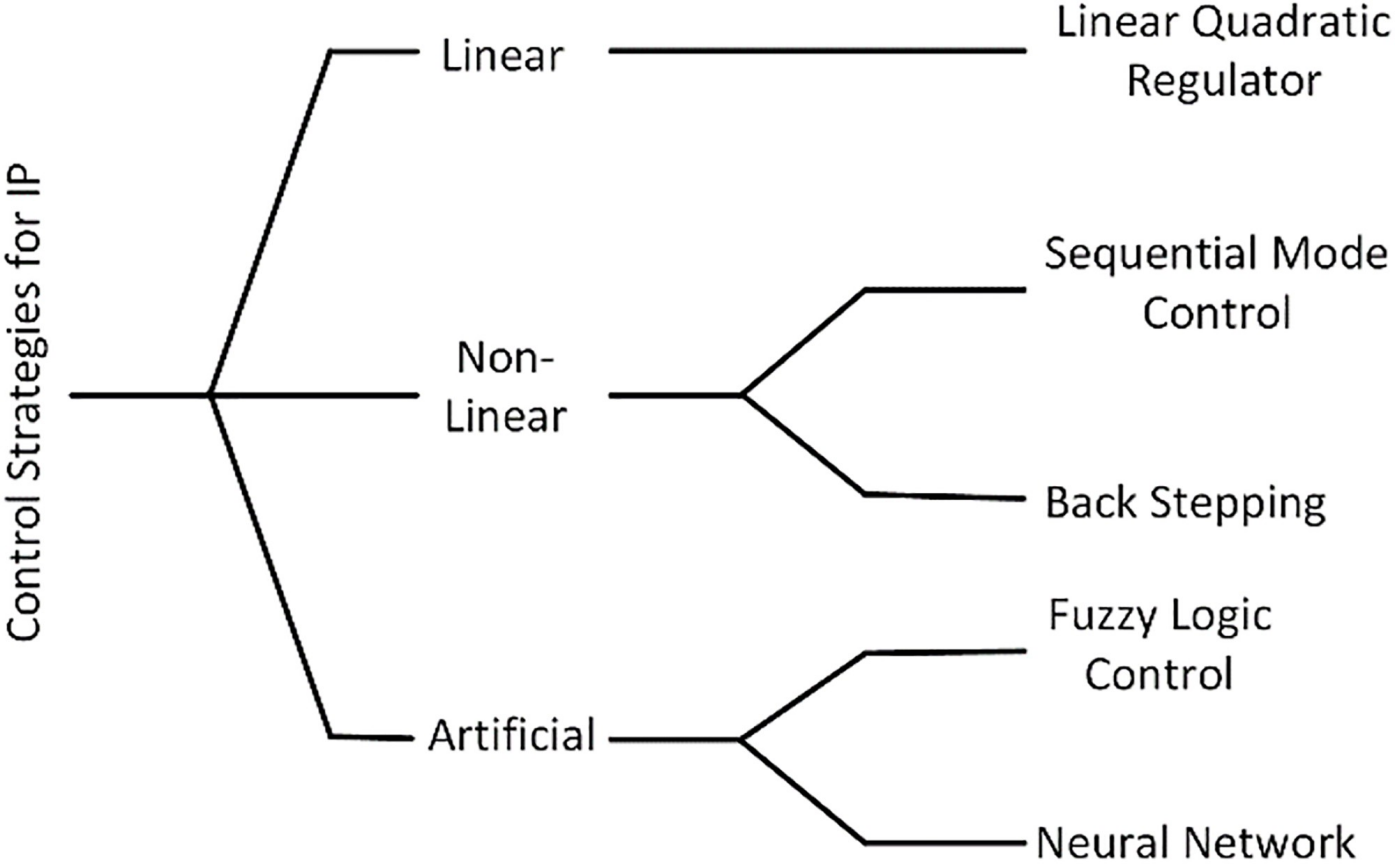
Control strategies for an IP.

This section describes the implemented control techniques for IP. There are three categories of control techniques for IP, that is, linear, nonlinear, and AI control approaches, and some selected algorithms of each type are shown in Fig 2. LQR is briefly described as a linear control plan for IP, whereas SMC and BS are presented in the domain of nonlinear algorithms. Finally, AI control techniques such as FLC and SMCNN are explored.

### 3.1 Linear quadratic regulator

The LQR is an optimal linear control technique that can be effectively used to improve the overall performance of a linear system. In general, it is appropriate for linear systems where disturbances and uncertainties are not present [23]. The main objective of using the LQR is to estimate the gain that minimizes the cost function [24]. A quadratic function-based cost function is used, which is usually defined as:

$$\begin{array}{c} J=\int_{0}^{\infty }\left[x^{T}\left(t\right)Q\left(t\right)x\left(t\right)+u^{T}\left(t\right)R\left(t\right)u\left(t\right)\right]dt \end{array}$$ (5)

where *u* and *x* are the inputs and states of the system, respectively. Similarly, *R* and *Q* are the positive definite matrices, where *Q*(*t*) ∈ *R*^*n*×*n*^ is a positive definite or positive semi-definite Hermitian matrix and *R*(*t*) ∈ *R*^*r*×*r*^ is a positive definite Hermitian matrix (or real constant number). The gain of the LQR is calculated using the following equation:

$$\begin{array}{c} K=R^{-1}B^{T}P \end{array}$$ (6)

The following equation gives the Riccati equation for finding the gain of the system

$$\begin{array}{c} A^{T}P+PA-PBR^{-1}B^{T}P+Q=0 \end{array}$$ (7)

Similarly, the generalized illustration of a linear control system is given by

$$\begin{array}{c} \dot{x}\left(t\right)=Ax\left(t\right)+Bu\left(t\right) \end{array}$$ (8)

Whereas the control law for such a linear system is defined as

$$\begin{array}{c} u(t)=-Kx(t) \end{array}$$ (9)

To compute the gains of the system, the system’s open loop response is incorporated and given by

$$\begin{array}{c} \dot{x}\left(t\right)=Ax\left(t\right)-BKx\left(t\right)=\left(A-BK\right)x\left(t\right) \end{array}$$ (10)

### 3.2 Sliding mode control

**Fig 3:**
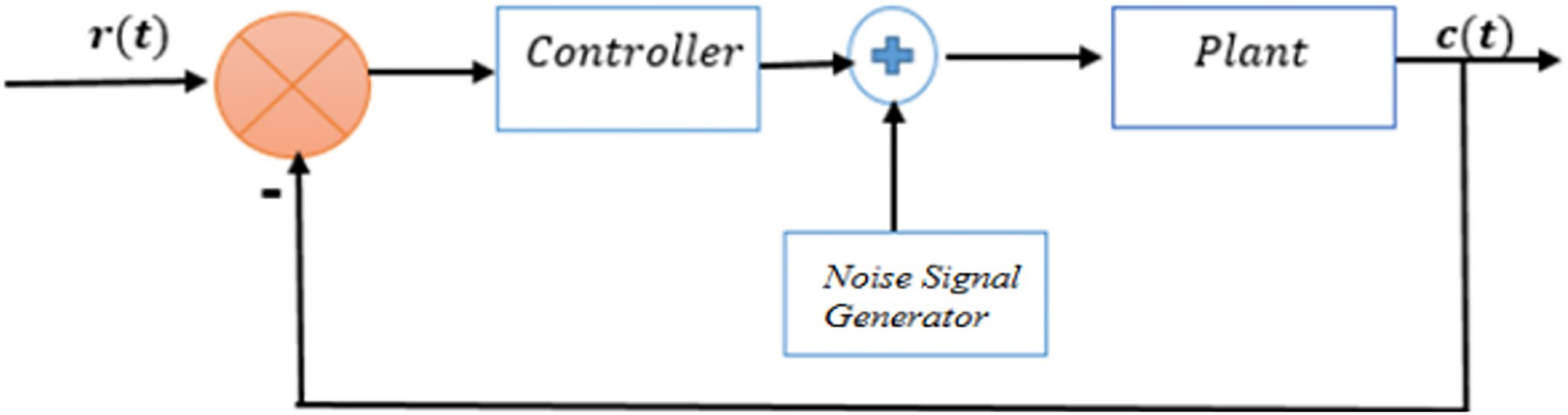
Block diagram of overall controller part of SMC.

SMC is a robust control technique capable of handling systems with multiple inputs and outputs (MIMO). However, there is always a need for more consistency between the actual model and the mathematical model of the plant when designing the controller [25]. Matched and unmatched uncertainties, external disturbances, and parametric uncertainties are the main inconsistencies between the actual and mathematical models of the plant. A robust controller is required to reduce these fundamental factors. SMC ensures global stability and effectively handles the fast dynamic response of a system [26]. A block diagram of the overall design is shown in Fig 3.

**Fig 4:**
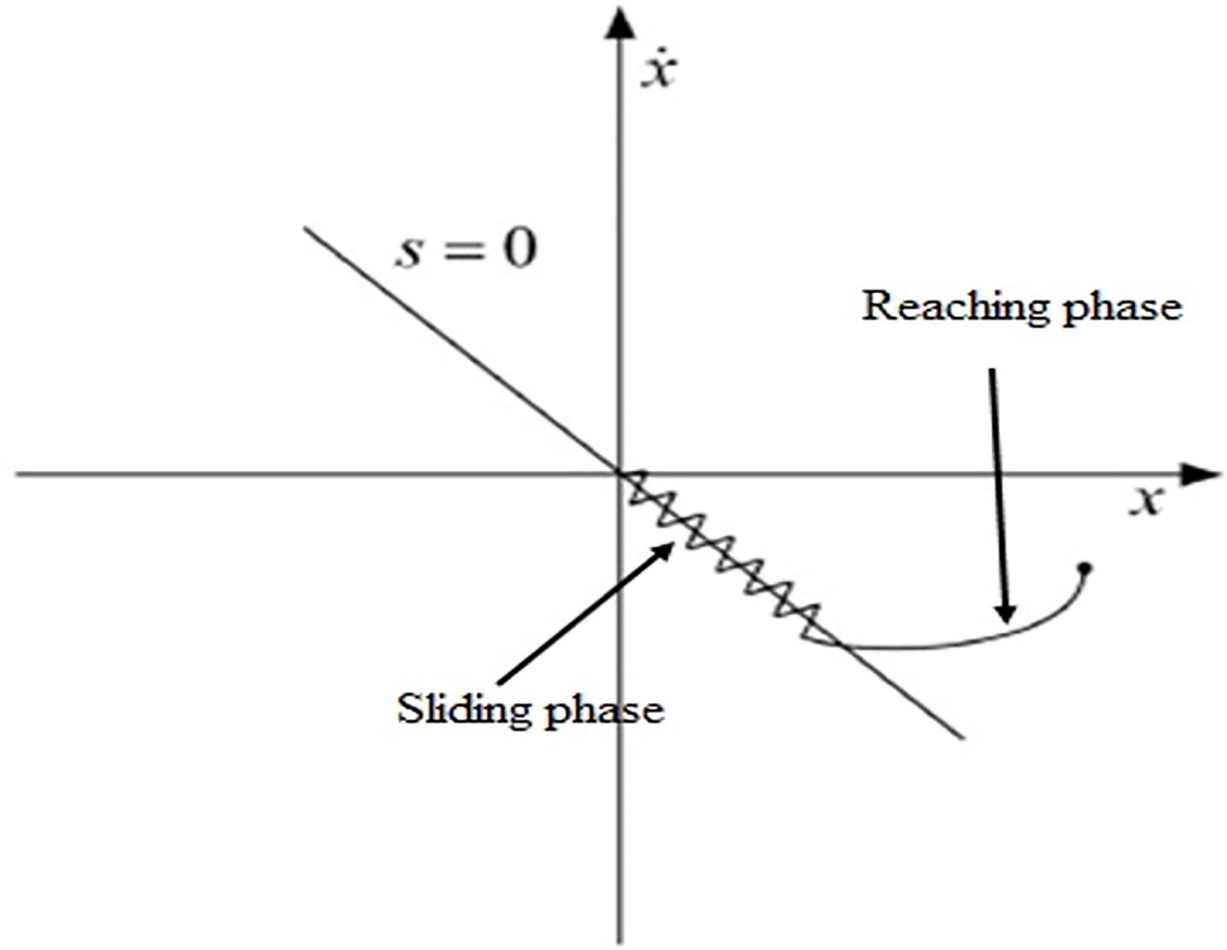
Block phases of SMC.

SMC consists of two phases: sliding and reaching. In the reaching phase, the system moves from its initial stage to the final desired trajectories, whereas in the sliding phase, the system remains there at all times. We have two parts of the SMC controller: an equivalent controller and a discontinuous controller. An equivalent controller is designed for the reaching phase, whereas a discontinuous controller is suitable for the sliding surface. The addition of these two controllers resulted in an overall controller for the system. The reaching and sliding phases are illustrated in Fig 4.

#### 3.2.1 Control design with SMC

The control design of the SMC involves the following steps. First, the sliding surface is defined by the following equation

$$\begin{array}{c} S\left(x,t\right)={\left(d/dt+\lambda \right)}^{3}e \end{array}$$ (11)

Where, *e* is the error signal and λ is the positive constant

$$\begin{array}{c} e=e_{1}-e_{d} \end{array}$$ (12)

$$\begin{array}{c} s=e^{3}+\lambda^{3}e+3\lambda^{2}\dot{e}+3\lambda \ddot{e} \end{array}$$ (13)

Then, by differentiating the sliding surface with respect to time and forcing the time derivative at $\left(s\right)=\dot{0}$, we get the equivalent controller part of SMC as

$$\begin{array}{c} u_{eq}=\left(\left(f\left(x\right)+\left(3\lambda e_{4}\right)+\left(3\lambda^{2}e_{3}\right)+\left(\lambda^{3}e_{2}\right)\right)/g\left(x\right)\right)+(\left({\dddot{e}}_{d}+\left(3\lambda^{2}{\ddot{e}}_{d}\right)+\left(3\lambda {\dddot{e}}_{d}\right)+\left(\lambda^{3}{\dot{e}}_{d}\right)\right)/g\left(x\right) \end{array}$$ (14)

While the discontinuous controller is designed by using a pre-defined sign function given by

$$\begin{array}{c} u_{dis}=-ksign\left(s\right) \end{array}$$ (15)

The overall controller is obtained through the addition of equivalent and discontinuous parts given as

$$\begin{array}{c} u=u_{eq}+u_{dis} \end{array}$$ (16)

Owing to these huge benefits, the first-order SMC suffers from the chattering phenomenon due to oscillation from the frequency turbulence. Higher-order Sliding Mode Control (HOSMC) is proposed to reduce the chattering effect [27]. The Lyapunov stability theorem is used to ensure the stability of the IP system. It is defined as follows,

$$\begin{array}{c} V=1/2S^{2} \end{array}$$ (17)

$$\begin{array}{c} \dot{V}=S\dot{S} \end{array}$$ (18)

By substituting the time derivative of Eq (17) into the above equation, the derivative of the Lyapunov function $\dot{V}$ is obtained:

$$\begin{array}{c} \dot{V}=S\left(-ksign\left(s\right)\right) \end{array}$$ (19)

$$\begin{array}{c} \dot{V}\leq -k\left|s\right| \end{array}$$ (20)

$\dot{V}$ becomes negative definite and the system dynamics converge to its origin in finite time.

### 3.3 Back-stepping control

The BS is a robust control technique that is highly nonlinear and based on the Lyapunov stability theorem. Stability is achieved through the recursive process, as Lyapunov is a scalar function that ensures the stability of the system [28]. This technique can only be implemented by using strict feedback systems. Because the IP is an underactuated system, rather than a pure feedback system, we cannot apply back-stepping directly to the IP. After transformation into the feedback linearizable form, we can apply the BS to the IP control problem. The IP swung initially and stabilized the upright position. The swung-up is obtained through a nonlinear controller, whereas the linear control stabilizes its dynamics. Linearized control techniques stabilize the angle and cart position using transformed regulated variables [29].

#### 3.3.1 Control design with BS

The control design of the BS method has a Lyapunov Function (LF), and a virtual signal is constructed to stabilize the subsystem until the signal enters system dynamics. Let the first tracking error for the cart position and pendulum’s angular position (*θ* = *x*_3_) be given as

$$\begin{array}{c} e_{1}=x_{1d}-x_{1} \end{array}$$ (21)

$$\begin{array}{c} e_{3}=x_{3d}-x_{3} \end{array}$$ (22)

where *x*_1*d*_ is the desired trajectory of the cart position and *x*_3*d*_ is the desired trajectory of the pendulum’s angular position. The stabilizing functions (*α*_1_, *α*_2_) are defined as follows.

$$\begin{array}{c} \alpha_{1}\left(x,e\right)={\dot{x}}_{1d}+k_{1}e_{1} \end{array}$$ (23)

$$\begin{array}{c} \alpha_{2}\left(x,e\right)={\dot{x}}_{3d}+k_{3}e_{3} \end{array}$$ (24)

Lyapunov candidate functions are defined in terms of the four regulatory variables *e*_1_, *e*_2_, *e*_3_ and *e*_2_ as follows

$$\begin{array}{c} V_{1}\left(e_{1},e_{2}\right)=1/2\left(e_{1}^{2}+e_{2}^{2}\right) \end{array}$$ (25)

$$\begin{array}{c} V_{2}\left(e_{3},e_{4}\right)=1/2\left(e_{3}^{2}+e_{4}^{2}\right) \end{array}$$ (26)

Considering the transformed regular form of the cart-pendulum system [30], virtual control input *tan*(*x*_3_) and system control input *u* are chosen to stabilize *x*_1_ and *x*_3_, respectively.

$$\begin{array}{c} x_{3}={\text{tan}}^{-}1\left(1/\left[g/c\left(\left(4/3-{\text{cos}}^{2}\;x_{3}\right)+4/3M\right)+4/3\left(lx_{4}^{2}\right)/\;\text{cos}\;x_{3}\right]\left(e_{1}+{\ddot{x}}_{1d}-k_{1}\left(e_{2}+k_{1}e_{1}\right)k_{2}e_{2}\right)\right) \end{array}$$ (27)

$$\begin{array}{c} u=-1/\;\text{cos}\;x_{3}\left(1/cl\left(e_{3}k_{4}e_{4}+{\ddot{x}}_{3d}-k_{3}\left(e_{4}+k_{3}e_{3}\right)\right)-\left(M+m\right)g\;\text{sin}\;x_{3}\right)-mlx_{4}^{2}\;\text{sin}\;x_{3} \end{array}$$ (28)

The virtual control input *tanx*_3_ and system control input *u* satisfy ${\dot{V}}_{1}\left(e_{1},e_{2}\right)<0$ and ${\dot{V}}_{2}\left(e_{3},e_{4}\right)<0$, respectively. Where *k*_1_, *k*_2_, *k*_3_, *k*_4_ > 0.

$$\begin{array}{c} {\dot{V}}_{1}\left(e_{1},e_{2}\right)=-k_{1}e_{1}^{2}-k_{2}e_{2}^{2} \end{array}$$ (29)

$$\begin{array}{c} {\dot{V}}_{2}\left(e_{3},e_{4}\right)=-k_{3}e_{3}^{2}-k_{4}e_{4}^{2} \end{array}$$ (30)

### 3.4 Fuzzy-logic controller

FLC is a model-less control technique that is suitable for systems with nonlinearities and disturbances [31]. This is an AI control technique that stabilizes the behavior of nonlinear systems. FLC is a challenging task, especially when using the IF-Then rule. This will work like human intelligence, and the accuracy of the control action will also increase [32]. FLC is an effective technique that systematically controls an uncertain system such as IP [33].

**Fig 5:**
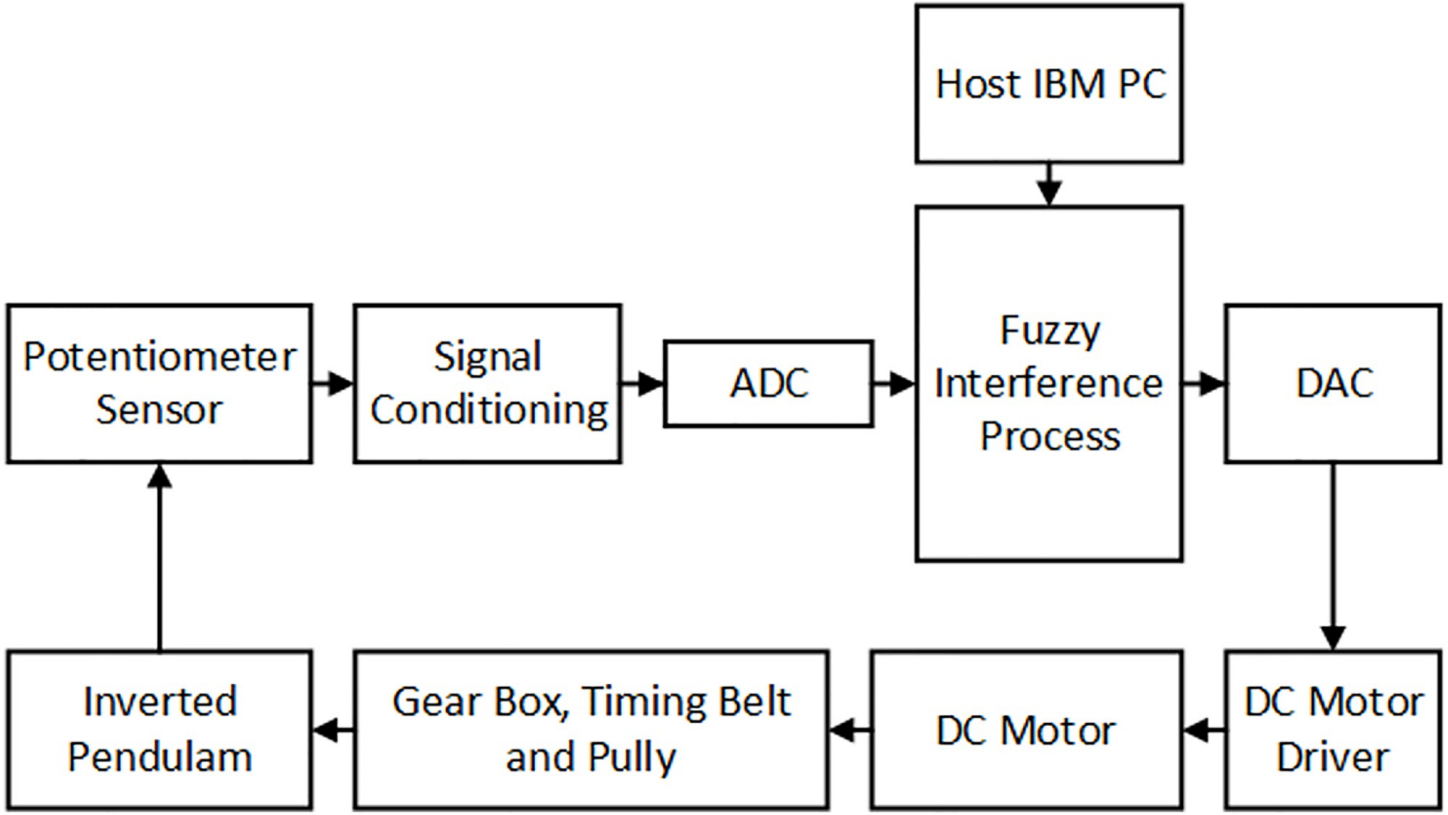
Diagram for the fuzzy control of the inverted pendulum [27].

Fig 5 shows an FLC block diagram in which the fuzzy interference process defines the logic to control the DC motor of the IP system through a DAC and amplifier. The microcontroller performs control actions. It samples the input data from the sensors and then controls the speed of the DC motor. The DC motor moves the cart position and balances IP [34]. A control design using a fuzzy logic technique is required to obtain the accuracy and stability of the system. The fuzzy interference rules for IP are mainly derived through Mamdani [35] and Sugeno techniques [36]. The basic steps of a fuzzy logic control design are the following:

1. Define fuzzy interface process
2. Define the input, output, and membership function
3. Use the IF-Then rule

There are four inputs and two outputs of the fuzzy controller for the IP. Two membership functions are assigned to each input variable. The Sugeno systems contain 16 output variables. We have 16 Sugeno IF-Then rules for IP. A few important rules are given below:

1. (Position is PL) and (Velocity is VL) and (angle is AL) and (angular velocity is AVL) then (Force is FLM),
2. (Position is PL) and (Velocity is VL) and (angle is AL) and (angular velocity is AVL) then (Force is FL),
3. (Position is PL) and (Velocity is VL) and (angle is AL) and (angular velocity is AVL) then (Force is zero),
4. (Position is PH) and (Velocity is VL) and (angle is AL) and (angular velocity is AVH) then (Force is zero),
5. (Position is PH) and (Velocity is VL) and (angle is AL) and (angular velocity is AVH) then (Force is TR),
6. (Position is PH) and, (Velocity is VL) and, (angle is AL), and (angular velocity is AVH), then (Force is TRM).

### 3.5 SMC-based neural network

The SMCNN is a nonlinear control technique. Owing to its lack of stability and nonlinearity, it provides a path for testing the prototype controller. Therefore, different researchers have designed NN-based controllers to test IP [37, 38]. A supervised NN reduces errors more efficiently and keeps the system stable [39, 40]. The RBF does not require mathematical modeling and can identify nonlinear and complex systems. A SMCNN can successfully track both IP axes more accurately and effectively [20, 41]. The Gyroscopic Inverted Pendulum (GIP), which is both nonlinear and unstable in an open loop, is evaluated on a single-layered NN with a nonlinear autoregressive moving average property [21].

#### 3.5.1 Control design

**Fig 6:**
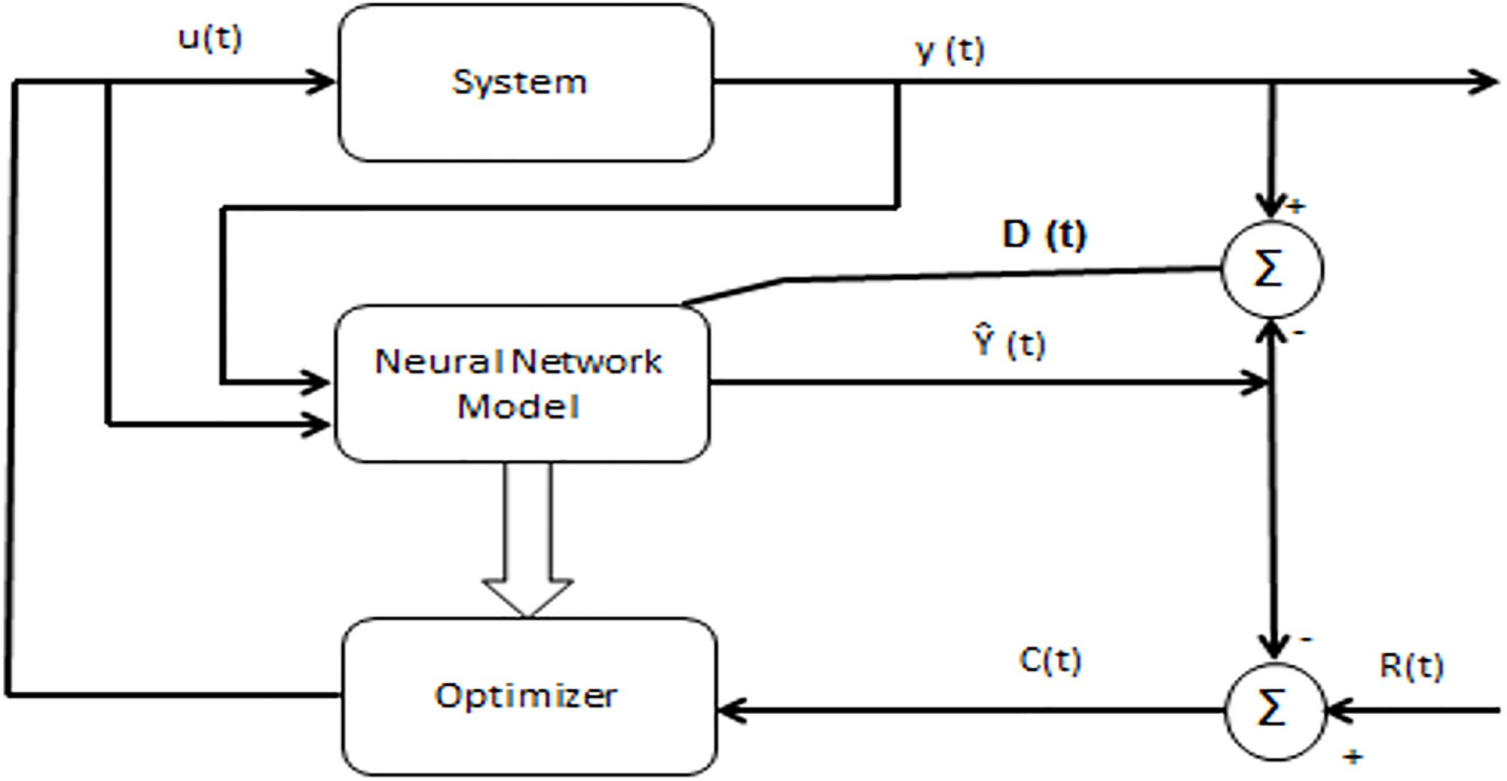
Neural network controller [40].

Fig 6 shows the overall closed-loop control system structure comprising an RBF NN, which estimates *F*(*x*) and the controller realized by an optimizer. To approximate the uncertain *F*, RBF networks are used adaptively. The algorithm of RBF networks is given by

$$\begin{array}{c} p_{j}=g\left(\|x-c_{ij}{\|}^{2}/\left(b_{j}^{2}\right)\right) \end{array}$$ (31)

$$\begin{array}{c} F=Q^{T}p\left(x\right)+r \end{array}$$ (32)

where *x* represents the network’s input state, *i* counts the input number of the network, *j* is the number of hidden layer nodes in the network, while *p* = [*p*_1_ *p*_2_ *p*_3_ … *p*_*n*_]^*T*^ is the yield of Gaussian function, *Q* is a vector of weights of the specified NN, *r* is approximation error of NN, and *r* ≤ *r*_*N*_. RBF network approximation *f* is used. The network input is chosen as $x={\left[e\;\;\dot{e}\right]}^{T}$, and the output of RBF neural network is

$$\begin{array}{c} \hat{F}={\hat{Q}}^{T}p\left(x\right) \end{array}$$ (33)

where *p*(*x*) is the NN’s Gaussian function in general and the Gaussian function parameters and neural network weights are difficult to choose. For this purpose, an error signal is defined as

$$\begin{array}{c} e_{1}=x_{3d}-x_{3} \end{array}$$ (34)

A sliding manifold is given by

$$\begin{array}{c} s={\dot{e}}_{1}+\lambda e_{1} \end{array}$$ (35)

Differentiating (35),

$$\begin{array}{c} \dot{s}={\ddot{e}}_{1}+\lambda {\dot{e}}_{1}={\ddot{x}}_{3d}-F\left(x\right)-G\left(x\right)u-D\left(t\right)+\lambda {\dot{e}}_{1} \end{array}$$ (36)

The control input is as follows

$$\begin{array}{c} u=\frac{1}{g}\left(-\hat{F}\left(x\right)+{\ddot{x}}_{3d}+\lambda {\dot{e}}_{1}+k\;sgn\left(s\right)\right) \end{array}$$ (37)

Substituting Eqs (37) in (36), we have

$$\begin{array}{c} \dot{s}=-F\left(x\right)+\hat{F}\left(x\right)-k\;sgn\left(s\right)-D\left(t\right)=-\tilde{F}\left(x\right)-k\;sgn\left(s\right)-D\left(t\right) \end{array}$$ (38)

where,

$$\begin{array}{c} \tilde{F}\left(x\right)=F\left(x\right)-\hat{F}\left(x\right) \end{array}$$ (39)

$$\begin{array}{c} \tilde{F}\left(x\right)=Q^{T}p\left(x\right)+r-{\hat{Q}}^{T}p\left(x\right) \end{array}$$ (40)

$$\begin{array}{c} \tilde{F}\left(x\right)={\tilde{Q}}^{T}p\left(x\right)+r \end{array}$$ (41)

where,

$$\begin{array}{c} \tilde{Q}=Q-\hat{Q} \end{array}$$ (42)

Defining the Lyapunov function as

$$\begin{array}{c} V\left(s,Q\right)=\frac{1}{2}s^{2}+\frac{1}{2}\gamma {\tilde{Q}}^{T}\tilde{Q} \end{array}$$ (43)

where *γ* is the positive coefficient of the above equation. By taking the derivative of *V*, we get

$$\begin{array}{c} \dot{V}\left(s,Q\right)=s\dot{s}+\gamma {\tilde{Q}}^{T}\dot{\tilde{Q}} \end{array}$$ (44)

Substituting the right-hand side of $\dot{s}$ and $\dot{\tilde{Q}}$,

$$\begin{array}{c} \dot{V}\left(s,Q\right)=s\left(-\tilde{F}\left(x\right)-k\;sgn\left(s\right)-D\left(t\right)\right)-\gamma {\tilde{Q}}^{T}\dot{\hat{Q}} \end{array}$$ (45)

$$\begin{array}{c} \dot{V}\left(s,Q\right)=-s\left(r+k\;sgn\left(s\right)+D\left(t\right)\right)-\tilde{Q}Q^{T}\left(\gamma \dot{\hat{Q}}+s\;p\left(x\right)\right) \end{array}$$ (46)

The adaptive control law is designed as follows

$$\begin{array}{c} \dot{\hat{Q}}=\frac{1}{\gamma }s\;p\left(x\right) \end{array}$$ (47)

$$\begin{array}{c} \dot{V}\left(s,Q\right)=-s\left(r+k\;sgn\left(s\right)+D\left(t\right)\right)=-s\left(r+D\left(t\right)\right)-k\left|s\right| \end{array}$$ (48)

We obtain approximately $\dot{V}\left(s,Q\right)\leq 0$ because the approximation error *r* is sufficiently small in design *k* ≥ *r*_*N*_ + *D*. We obtain approximately $\dot{V}\left(s,Q\right)\leq 0$ because the approximation error *r* is sufficiently small in design *k* ≥ *r*_*N*_ + *D*. Table 1 presents a review of different properties of the studied algorithms for IP. Linear techniques such as LQR are simple to implement with less computational complexity than nonlinear techniques such as Artificial and non-artificial based control design. However nonlinear techniques show better transient and steady state performance.

**Table 1:** Limitations and advantages of different control stratifies, a comparison.

No.	Technique/ Algorithm	Dependencies	Limitations	Performance
1	Linear Quadratic Regulator	Linearization	Only for linear systems	Robust
2	Sliding Mode Control	Feedback linearization	Chattering problem	Robust
3	Back Stepping	Feedback linearization	Robustness	Non-linear/ Adaptive
4	Fuzzy Logic Control	Linguist variables IF-Then rules	Difficult to implement	Robust
5	SMC based Neural Network	Dataset, Network Architecture	Large dataset required to train the system	Adaptive

## 4 Results and discussion

In this section, we delve into the assessment and comparison of control algorithms, focusing on selecting a representative algorithm from each of the three categories. These selections are made based on the criteria of low complexity and reasonable accuracy when compared to other strategies within their respective classes. Our analysis encompasses key performance parameters such as settling time, rise time, steady-state percentage error, and percentage overshoot. The results for each parameter are presented in a step-wise manner below.

**Fig 7:**
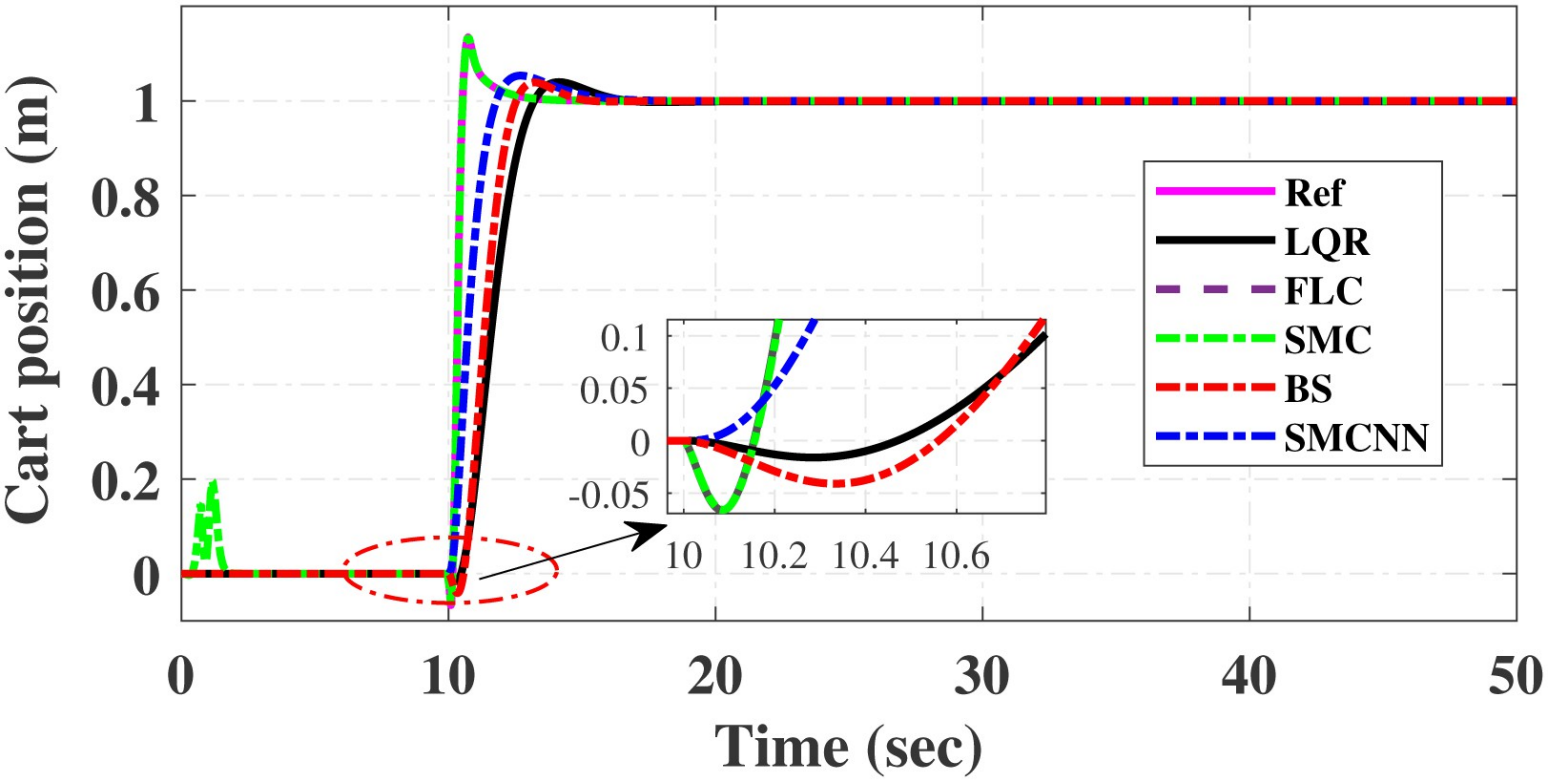
Comparative results of step response of cart-position.

**Fig 8:**
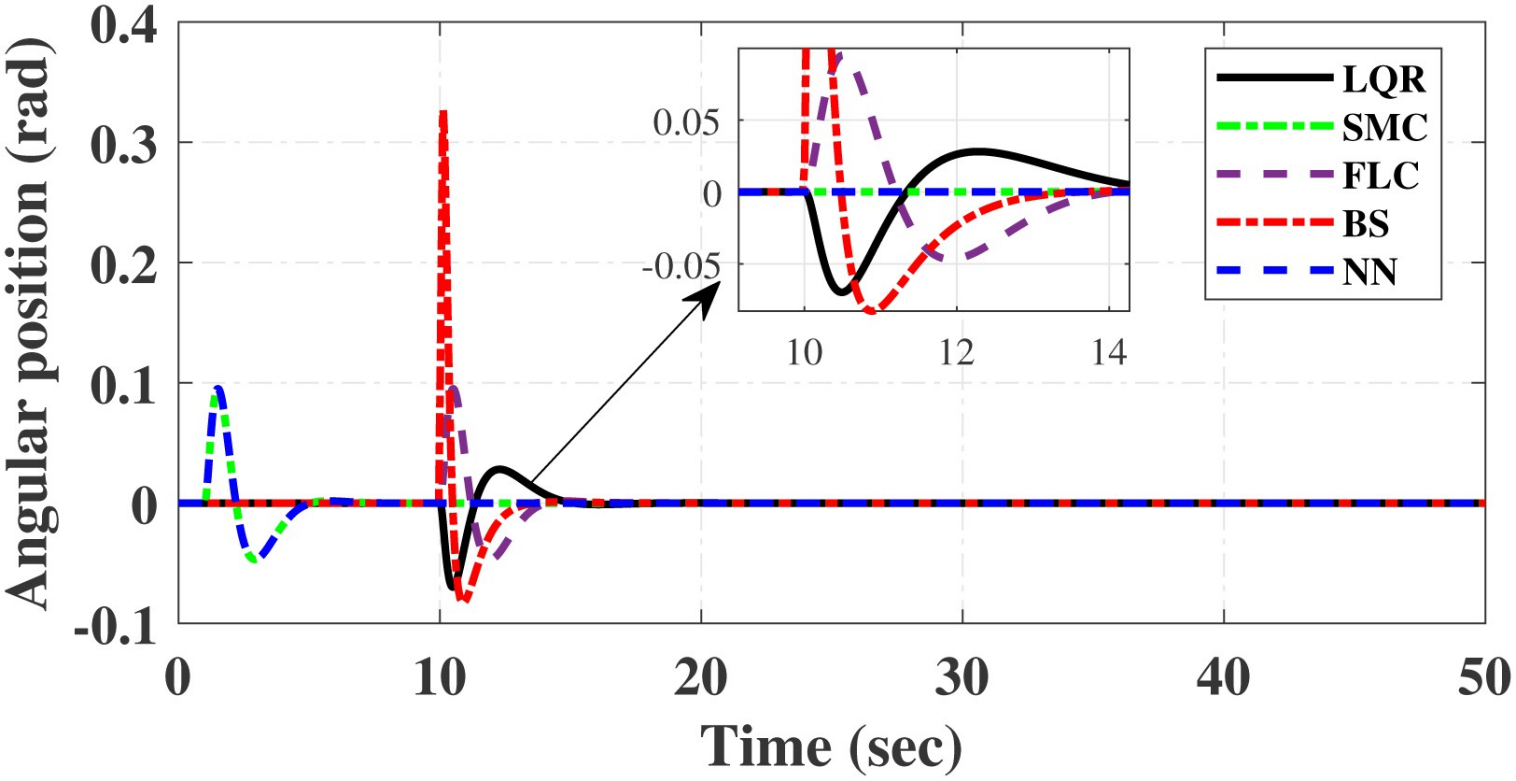
Comparative results of step response of pendulum.

**Fig 9:**
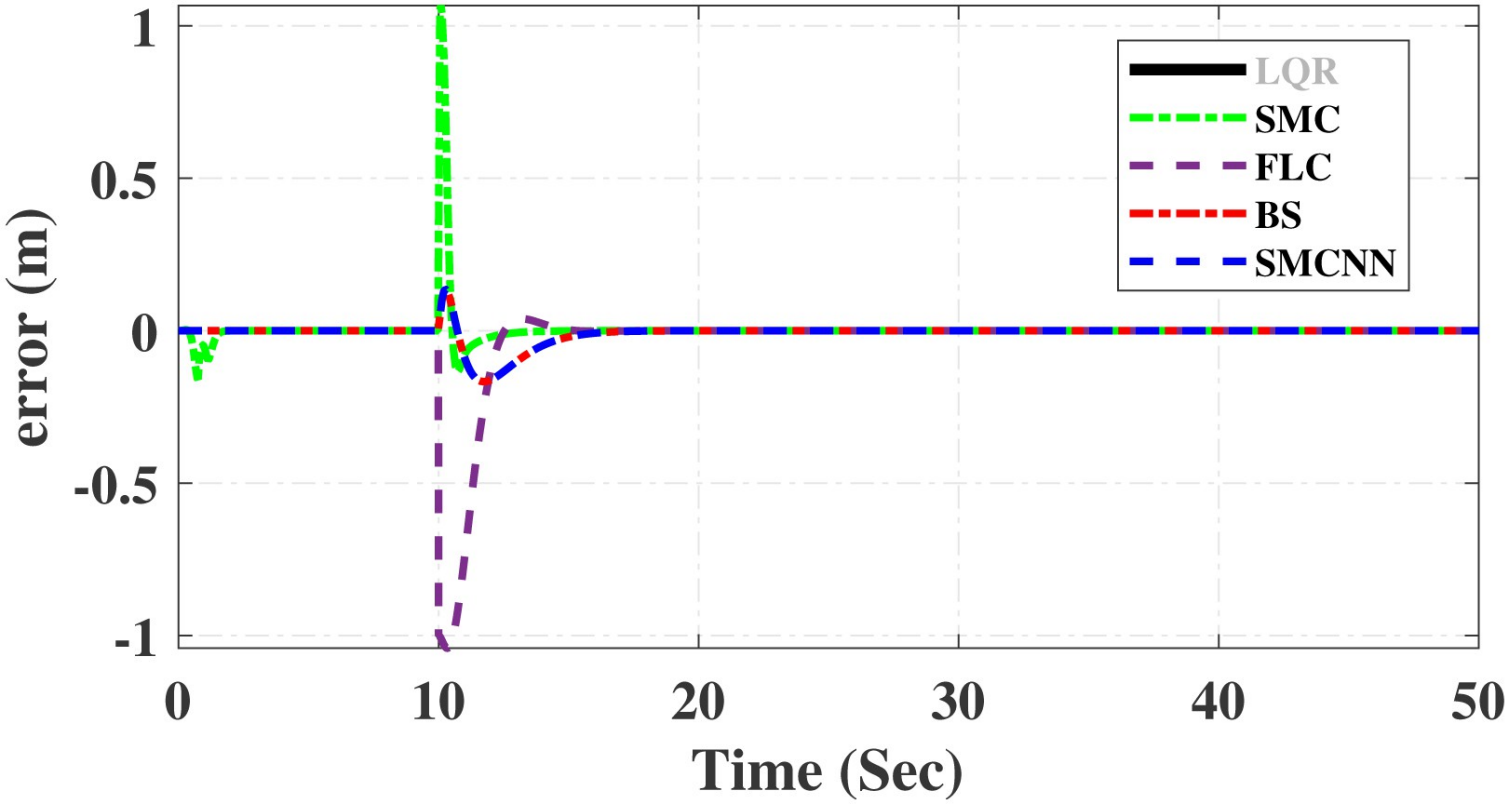
Comparative results of cart’s position error signals.

**Fig 10:**
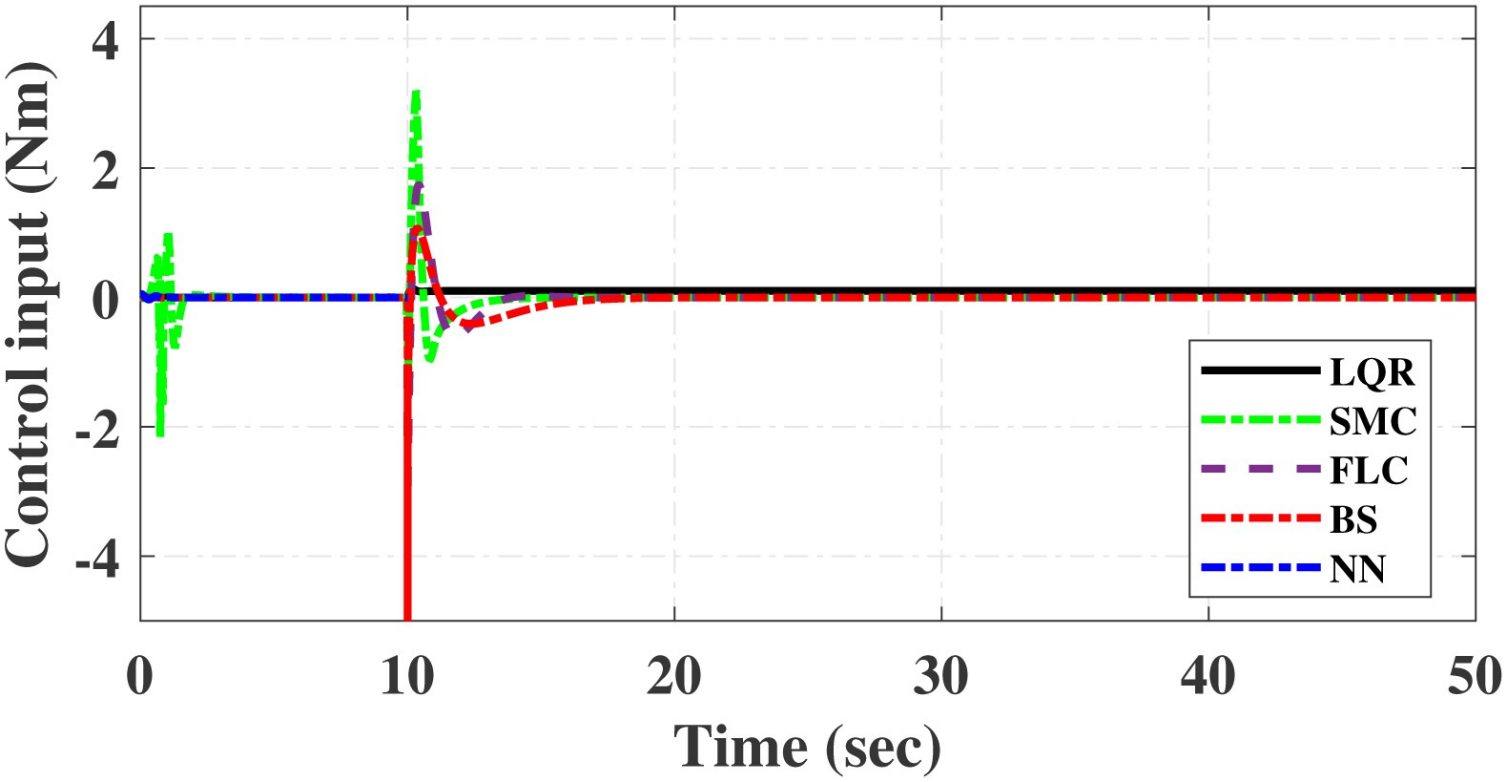
Comparative results of control inputs.

First, Figs 7 and 8 scrutinize the outcomes obtained from all controllers concerning the cart’s position and the pendulum’s angle. The findings indicate that the system achieves stability in both the cart’s position and pendulum’s angle after the settling time. Notably, given the inverted pendulum’s non-minimum phase nature, the cart initially moved in the opposite direction before successfully tracking the desired position. To provide a comprehensive view of performance, Table 2 details the efficacy of simulated control techniques across all algorithms. Turning our attention to Figs 7 and 8, we examine the response of the cart position and pendulum angle to a step input. Remarkably, all control algorithms effectively restored equilibrium in the cart position and pendulum, achieving upright stability after specific settling times. For instance, the SMCNN control technique emerges as particularly efficient, stabilizing the cart position in a mere 1.5 seconds, outperforming the other strategies in this regard. Similarly, the pendulum angle swiftly converges to its desired trajectory when the SMCNN control technique is applied. Figs 9 and 10 provide further insights into the control inputs and tracking errors for the LQR, SMC, FLC, BS, and SMCNN strategies. In Fig 9, we observe a distinct chattering effect in the case of SMC, which is notably mitigated by the remaining techniques. Meanwhile, Fig 10 illustrates that the tracking error approaches near-zero values, showcasing the robustness of the simulated control strategies against both matched and unmatched uncertainties.

**Table 2:** Comparison of implemented control techniques for cart’s position and pendulum’s angle.

Specifications	LQR	SMC	FLC	BS	SMCNN
Settling time (sec)	6.2	3	5	1.5	4
Rise time (sec)	3.2	0.9	2.5	2	1.4
Steady-state error	0.02	0.0012	1	0	0
Overshoot	0.01	0.001	0.001	0	0

**Fig 11:**
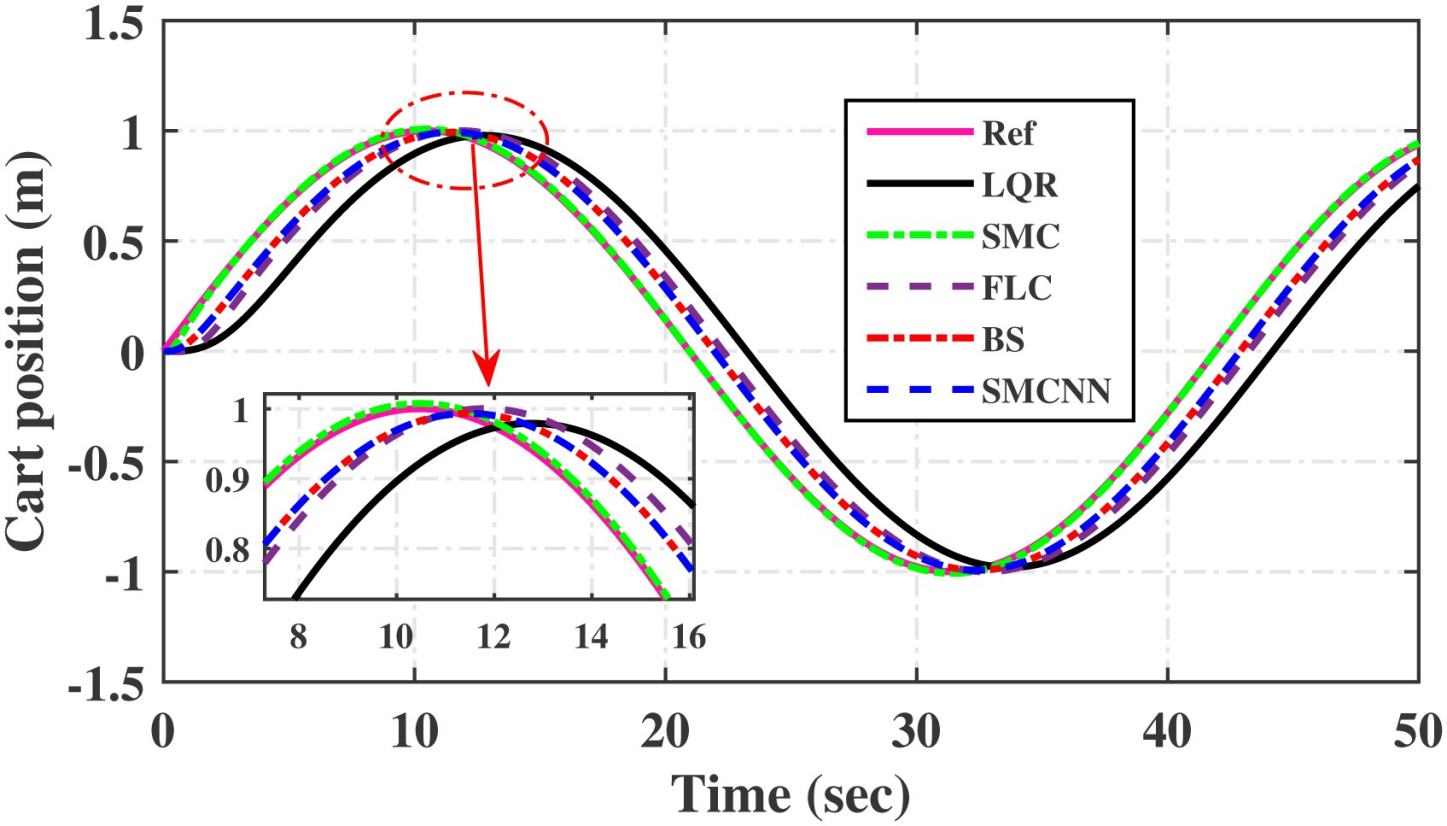
Simulated results of the sinusoidal response of cart-position.

**Fig 12:**
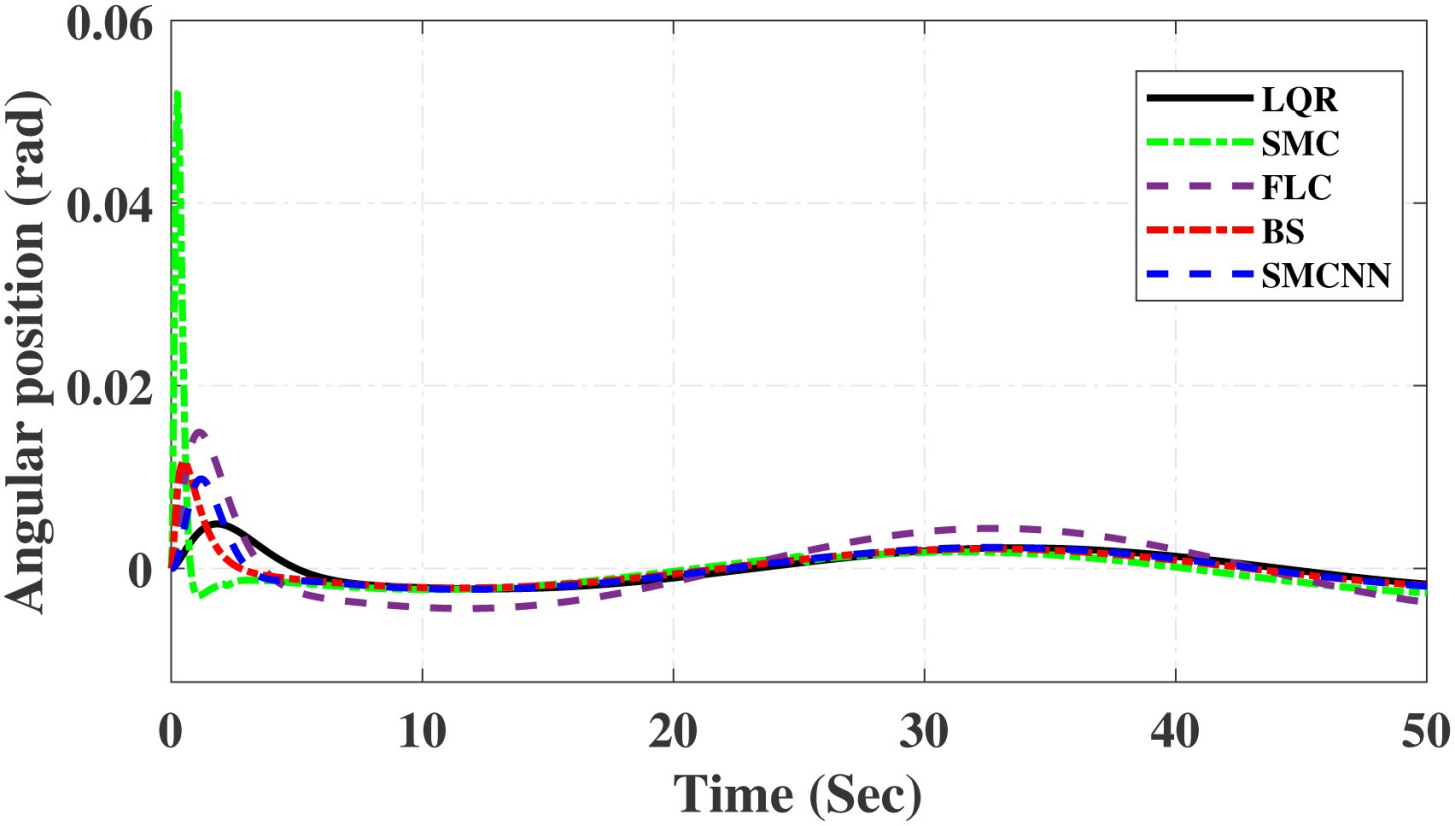
Simulated results of sinusoidal response of pendulum.

**Fig 13:**
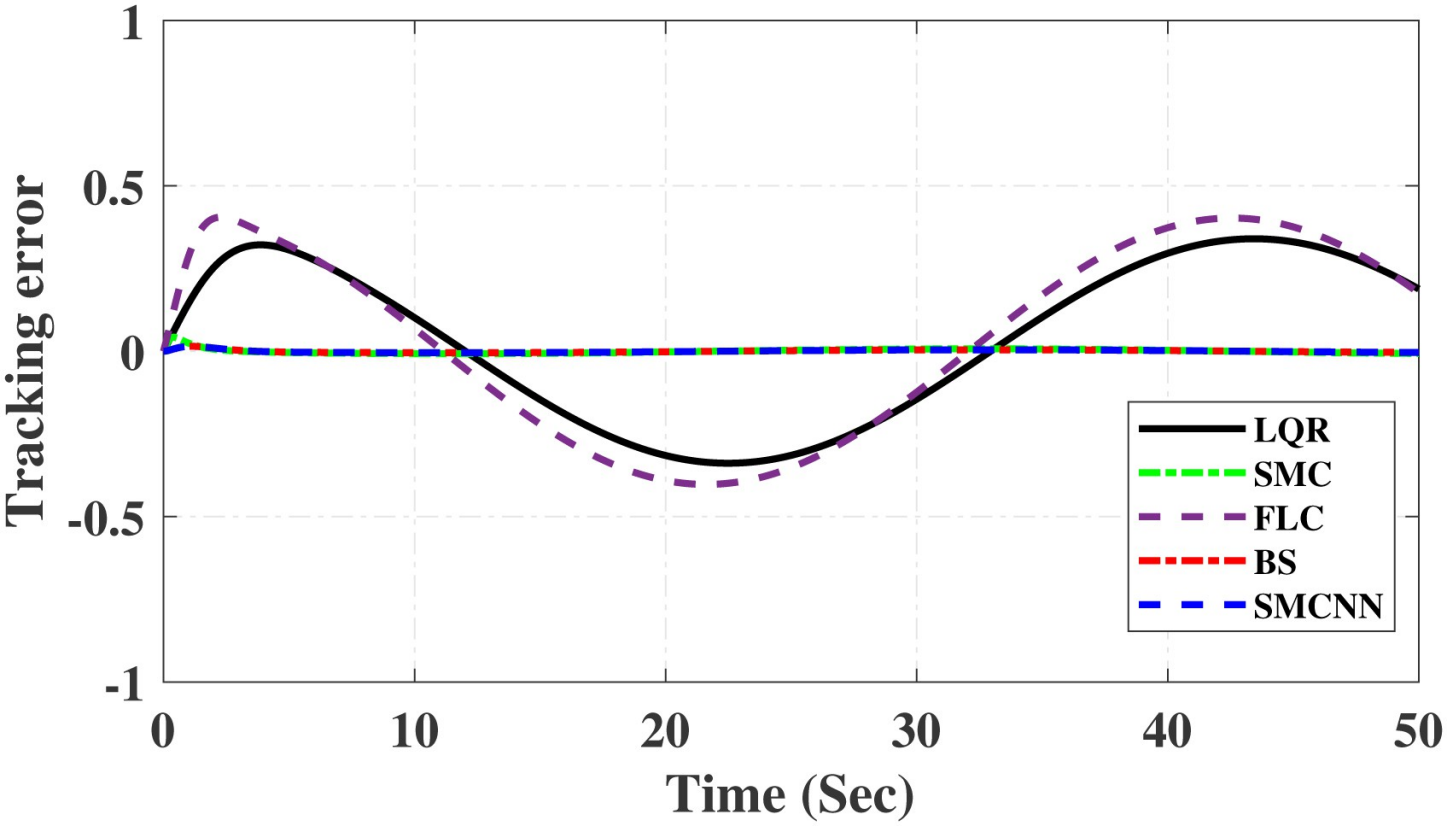
Simulated results of cart’s position error signals.

**Fig 14:**
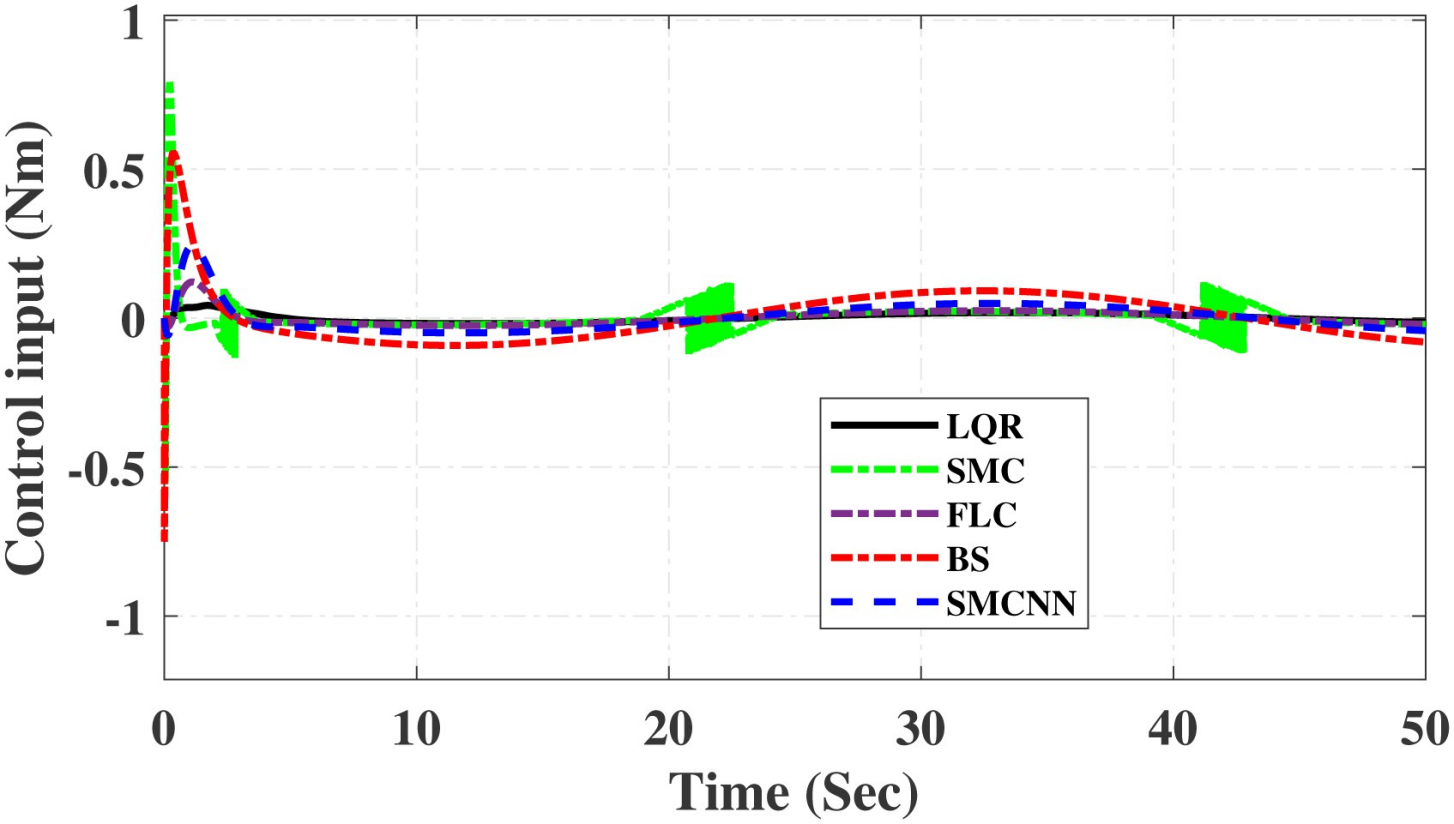
Simulated results of control inputs.

Simulated results for the cart position and pendulum angle under sinusoidal input conditions are depicted in Figs 11 and 12. These results provide valuable insights into the performance of all the controllers. Notably, stability is achieved in both the cart position and pendulum angle, with distinct settling times observed for each control algorithm: 6.2, 3, 5, 1.5, and 4 s for LQR, SMC, FLC, BS, and SMCNN, respectively. The pendulum exhibits an oscillatory motion in line with the desired trajectory of 2*π*. Fig 11 illustrates the cart’s movement towards the desired trajectory and its stabilization for all the control techniques. Figs 13 and 14 present the simulation results for the control input and error signals across all the simulated methods. Notably, the tracking error approached zero, demonstrating the effectiveness and robustness of the control strategies against both matched and unmatched uncertainties. Additionally, the chattering effect, particularly visible in the SMC case, is notably reduced when employing the BS and SMCNN techniques. A comprehensive overview of the controller performance can be found in Table 2.

In summary, the results obtained from these simulations indicate that all selected control algorithms exhibit stability and promising performance in achieving the desired system design goals.

## 5 Conclusions

In this research, we have demonstrated the implementation of a diverse range of control techniques, encompassing linear, nonlinear, and artificial intelligence-based approaches, to tackle real-time challenges. Our primary focus is on the inverted pendulum, which serves as a representative example of a nonlinear, underactuated mechanical system. The versatility of the IP concept extends to various cutting-edge applications, including space satellite control, aircraft landing systems, humanoid robot stability, seismometer control, and the balancing of ships against tides. Throughout this study, we have effectively harnessed robust control techniques to ensure stability and precise response in nonlinear systems, particularly emphasizing the challenging domain of IP control. Our research has brought forth a selection of control strategies tailored for IP control, namely FLC, SMCNN, SMC, and BS. Upon conducting a thorough comparative analysis, it becomes evident that the SMCNN controller emerges as the standout performer in critical aspects such as settling time, overshoot, and steady-state error. In this context, our findings showcase the superior capabilities of AI-based control approaches, exemplified by NNs, in addressing real-time control challenges, including the intricacies of the inverted pendulum.

Recognizing the importance of more extensive statistical analysis for a comprehensive evaluation of control strategies, future endeavors will involve the incorporation of more in-depth statistical analysis to further augment the findings and address this aspect in greater detail. This future work will contribute to a broader understanding of control systems in complex and dynamic environments.

## References

[1] Singh VK, Kumar V. Adaptive backstepping control design for stabilization of inverted pendulum. In: Students Conference on Engineering and Systems (SCES), May 28, 2014; pp. 1-5.

[2] Ullah S, Khan Q, Mehmood A, Bhatti AI. Robust backstepping sliding mode control design for a class of underactuated electro–mechanical nonlinear systems. Journal of Electrical Engineering & Technology. 2020 Jul;15(4):1821–8. doi: 10.1007/s42835-020-00436-3

[3] Ullah S, Khan Q. Mehmood A, Akmeliawati R., Integral backstepping based robust integral sliding mode control of underactuated nonlinear electromechanical systems. Journal of Control Engineering and Applied Informatics. 2019 Sep 25; 21(3): 42–50.

[4] Lee J, Mukherjee R, Khalil HK. Output feedback stabilization of an inverted pendulum on a cart in the presence of uncertainties. Automatica. Apr 30 2015; 54: pp. 146–57. doi: 10.1016/j.automatica.2015.01.013

[5] Lee MA, Takagi H. Integrating the design stage of fuzzy systems using genetic algorithms. In: Second IEEE International Conference on Fuzzy Systems, 1993; pp. 612-617.

[6] Cuevas PT, Luna AH, Sanchez JF, López IM, Bonilla SI. Stability of fuzzy and LQR controllers applied to an inverted pendulum system. In: Power, IEEE International Autumn Meeting on Electronics and Computing (ROPEC), Nov 4 2015; pp. 1-6.

[7] Reddy NS, Saketh MS, Pal P, Dey R. Optimal PID controller design of inverted pendulum dynamics: A hybrid pole-placement a firefly algorithm approach. In: IEEE First International Conference on Control, Measurement and Instrumentation (CMI), Jan 8 2016; pp. 305-310.

[8] Eltohamy KG, Kuo CY. Nonlinear optimal control of triple-link inverted pendulum with a single control input. International Journal of Control. Jan 1, 1998; 69(2): pp. 239–56. doi: 10.1080/002071798222811

[9] Li X, Ren Y. Design and improvement of fuzzy controller based on single inverted pendulum. 3rd International Conference on Management, Education, Information, and Control (MEICI), 2015; pp. 1671-1677.

[10] Munir M, Khan Q, Ullah S, Syeda TM, Algethami AA. Control Design for Uncertain Higher-Order Networked Nonlinear Systems via an Arbitrary Order Finite-Time Sliding Mode Control Law. Sensors. 2022 Apr 2;22(7):2748. doi: 10.3390/s22072748 35408362 PMC9003359

[11] Ajwad SA, Iqbal J, Khan AA, Mehmood A. Disturbance-observer-based robust control of a serial-link robotic manipulator using SMC and PBC techniques. Studies in Informatics and Control. Dec 1 2015; 24(4): pp. 401–408. doi: 10.24846/v24i4y201504

[12] Ullah S, Mehmood A, Ali K, Javaid U, Hafeez G, Ahmad E. Dynamic Modeling and Stabilization of Surveillance Quadcopter in Space based on Integral Super Twisting Sliding Mode Control Strategy. In2021 International Conference on Artificial Intelligence (ICAI) 2021 Apr 5 (pp. 271-278).

[13] Ullah S, Khan Q, Mehmood A, Kirmani SA, Mechali O. Neuro-adaptive fast integral terminal sliding mode control design with variable gain robust exact differentiator for under-actuated quadcopter UAV. ISA transactions. 2022 Jan 1;120:293–304. doi: 10.1016/j.isatra.2021.02.045 33771347

[14] Ullah S, Khan Q, Mehmood A. Neuro-adaptive fixed-time non-singular fast terminal sliding mode control design for a class of under-actuated nonlinear systems. International Journal of Control. 2023 Jun 3;96(6):1529–42. doi: 10.1080/00207179.2022.2056514

[15] Qiao WZ, Mizumoto M. PID type fuzzy controller and parameters adaptive method. Fuzzy sets and systems. Feb 26 1996; 78(1): pp. 23–35. doi: 10.1016/0165-0114(95)00115-8

[16] El-Bardini M, El-Nagar AM. Interval type-2 fuzzy PID controller for uncertain nonlinear inverted pendulum system. ISA transactions. May 1 2014; 53(3): pp. 732–743. doi: 10.1016/j.isatra.2014.02.007 24661774

[17] Wang H, Dong H, He L, Shi Y, Zhang Y. Design and simulation of the LQR controller with a linear inverted pendulum. In: International Conference on Electrical and Control Engineering (ICECE), Jun 25 2010; pp. 699-702.

[18] Anderson CW. Learning to control an inverted pendulum using neural networks. IEEE Control Systems Magazine. Apr 1989; 9(3): pp. 31–37. doi: 10.1109/37.24809

[19] Wu Q, Sepehri N, He S. Neural inverse modelling and control of a base-excited inverted pendulum. Engineering Applications of Artificial Intelligence. Jun 1 2002; 15(3-4): pp. 261–272. doi: 10.1016/S0952-1976(02)00042-8

[20] Mladenov V. Application of neural networks for inverted pendulum control WSEAS Transactions on Circuits and Systems. Feb 1 2011; 10(2): pp. 49–58.

[21] Pasemann F. Evolving neurocontrollers for balancing an inverted pendulum. Network: Computation in Neural Systems. Jan 1 1998; 9(4): 495–511. doi: 10.1088/0954-898X_9_4_006 10221576

[22] Pathak K, Franch J, Agrawal SK. Velocity and position control of wheeled inverted pendulum using partial feedback linearization. IEEE Transactions on robotics. Jun 2005; 21(3): pp. 505–513. doi: 10.1109/TRO.2004.840905

[23] Abonyi J, Babuska R, Szeifert F. Fuzzy modeling with multivariate membership functions: Gray-box identification and control design. IEEE Transactions on Systems, Man, and Cybernetics, Part B (Cybernetics). Oct 2001; 31(5): pp. 755–767. doi: 10.1109/3477.956037 18244840

[24] Nagib G, Gharieb W, Binder Z. Application of fuzzy control to a nonlinear thermal process. In: Proceedings of the 31st IEEE Conference on Decision and Control, 1992; pp. 1154-1159.

[25] Yu WS, Karkoub M, Wu TS, Her MG. Delayed output feedback control for nonlinear systems with two-layer interval fuzzy observers. IEEE Transactions on Fuzzy Systems. Jun 2014; 22(3): pp. 611–630. doi: 10.1109/TFUZZ.2013.2269693

[26] Popa DD, Craciunescu A, Kreindler L. A PI-Fuzzy controller designated for industrial motor control applications. In: IEEE International Symposium on Industrial Electronics (ISIE). Jun 30 2008; pp. 949-954.

[27] Kickert WJ, Mamdani EH. Analysis of a fuzzy logic controller. Fuzzy sets and systems. Jan 1 1978; 1(1): pp. 29–44. doi: 10.1016/0165-0114(78)90030-1

[28] Kim HJ, Joo YH, Park JB. Controller Design for Continuous-Time Takagi-Sugeno Fuzzy Systems with Fuzzy Lyapunov Functions: LMI Approach. Int. J. Fuzzy Logic and Intelligent Systems. Sep 2012; 12(3): pp. 187–192. doi: 10.5391/IJFIS.2012.12.3.187

[29] Jakubczyk B. On linearization of control systems. Bull. Acad. Polonaise Sci. Ser. Sci. Math. 1980; 28: pp. 517–22.

[30] Khan Q, Akmeliawati R, Bhatti AI, Khan MA. Robust stabilization of underactuated nonlinear systems: A fast terminal sliding mode approach. In: ISA transactions. 2017 Jan 1; 66: pp. 241–248. doi: 10.1016/j.isatra.2016.10.017 27884392

[31] Guo ZQ, Xu JX, Lee TH. Design and implementation of a new sliding mode controller on an underactuated wheeled inverted pendulum. Journal of the Franklin Institute. Apr 1 2014; 351(4): pp. 2261–2282. doi: 10.1016/j.jfranklin.2013.02.002

[32] Jedda O, Ghabi J, Douik A. Second order sliding mode control for inverted Pendulum. In: 12th International Multi-Conference on Systems, Signals & Devices (SSD), Mar 16 2015; pp. 1-5.

[33] Rantzer A, Johansson M. Piecewise linear quadratic optimal control. IEEE transactions on automatic control. Apr 2000; 45(4): pp. 629–637. doi: 10.1109/9.847100

[34] Ozana S, Pies M, Slanina Z, Hajovsky R. Design and Implementation of LQR controller for Inverted Pendulum by use of REX Control System. In: 12th International Conference on Control, Automation and Systems (ICCAS), Oct 17 2012; pp. 343-347.

[35] Tong S, Wang T, Li Y, Chen B. A combined Back-stepping and stochastic small-gain approach to robust adaptive fuzzy output feedback control. IEEE Transactions on Fuzzy Systems. Apr 2013; 21(2): pp. 314–327. doi: 10.1109/TFUZZ.2012.2213260

[36] Benaskeur A, Desbiens A. Application of adaptive Back-stepping to the stabilization of the inverted pendulum. In: IEEE Canadian Conference on Electrical and Computer Engineering, May 24, 1998; Vol. 1, pp. 113-116.

[37] Haq IU, Khan Q, Ullah S, Khan SA, Akmeliawati R, Khan MA, Iqbal J. Neural network-based adaptive global sliding mode MPPT controller design for stand-alone photovoltaic systems. Plos one. 2022 Jan 20;17(1):e0260480. doi: 10.1371/journal.pone.0260480 35051183 PMC8775327

[38] Alam Z, Khan Q, Khan L, Ullah S, Kirmani SA, Algethami AA. Certainty-equivalence-based sensorless robust sliding mode control for maximum power extraction of an uncertain photovoltaic system. Energies. 2022 Mar 10;15(6):2029. doi: 10.3390/en15062029

[39] Jung S, Kim SS. Control experiment of a wheel-driven mobile inverted pendulum using neural network. IEEE Transactions on Control Systems Technology. Mar 2008; 16(2): 297–303. doi: 10.1109/TCST.2007.903396

[40] Cho HT, Jung S. Neural network position tracking control of an inverted pendulum an XY table robot. In: IEEE/RSJ International Conference on Intelligent Robots and Systems (IROS). Oct 27, 2003; Vol. 2, pp. 1210-1215.

[41] Mladenov V, Tsenov G, Ekonomou L, Harkiolakis N, Karampelas P. Neural network control of an inverted pendulum on a cart. In: Proceedings of the 9th WSEAS International Conference on Robotics, Control and Manufacturing Technology; May 20 2009; (No. 9). pp. 112-120

